# Ultrasound imaging of patellar tendon thickness in elite sprint track cyclists and elite soccer players: An intra-rater and inter-rater reliability study

**DOI:** 10.1371/journal.pone.0270871

**Published:** 2022-07-05

**Authors:** Sebastian Klich, Aureliusz Kosendiak, Igor Krymski, Adam Kawczyński, Pascal Madeleine, Cesar Fernández-de-las-Peñas

**Affiliations:** 1 Department of Paralympic Sport, Wrocław University of Health and Sport Science, Wrocław, Poland; 2 Study of Physical Education and Sport, Wrocław Medical University, Wrocław, Poland; 3 Polish Cycling Federation, Pruszków, Poland; 4 Department of Health Science and Technology, Sport Sciences–Performance and Technology, Aalborg University, Aalborg, Denmark; 5 Department of Physical Therapy, Occupational Therapy, Rehabilitation and Physical Medicine, Universidad Rey Juan Carlos, Madrid, Spain; 6 Cátedra Institucional en Docencia, Clínica e Investigación en Fisioterapia: Terapia Manual, Punción Seca y Ejercicio Terapéutico, Universidad Rey Juan Carlos, Madrid, Spain; Universiti Malaya, MALAYSIA

## Abstract

The goal of our study was to investigate the relative and absolute intra-rater and inter-rater reliability of ultrasound assessment of patellar tendon (PT) thickness assessed over four locations, in track cyclists and soccer players. Fifteen male elite track cyclists and 15 male elite soccer players participated. Tendon thickness was measured over 4 locations placed at 5-10-15-20 mm inferior to the apex of the patella by two experienced examiners. Each examiner took two US images for the test measurements with a 10-min rest period. After a 30-min period, the subjects underwent a retest measurements that were also repeated 1-week after. A two-way analysis of variance revealed a significant group x location interaction on PT thickness for Examiner 1 (p = .001, η^2^ = .81) and Examiner 2 (p = 0.001, η^2^ = 0.78). Intra-rater reliability ranged from good to excellent (ICC_2,k_ ≥ 0.75), whereas inter-rater reliability was good (ICC_2,k_ ≥ 0.75) in both groups. Ultrasonographic assessment of PT was found to be a reliable method to assess tendon thickness. The middle location of the PT (corresponding to 15 and 20 mm) can be considered the most reliable spot to measure PT thickness. The PT thickness was larger among track cyclists than soccer players, with larger differences over the distal location (15 mm). Ultrasonographic assessment of PT was found to be a reliable method to assess tendon thickness. The middle location of the PT corresponding to 15 mm and 20 mm can be considered the most reliable area to measure PT thickness.

## Introduction

Ultrasound (US) imaging is a clinical and research tool that is widely used for injury prevention and diagnosis [1, 2]. Radiological imaging modalities, such as US, magnetic resonance imaging (MRI), and computed tomography (CT), are commonly used for visually assessing the musculoskeletal system. Kartus et al. [3] reported good reliability for the measurement of non-injured patellar tendon (PT) thickness in a cross-sectional study comparing US and MRI. Warden et al. [4] reported that US was more accurate than MRI in confirming the clinical diagnosis of patellar tendinopathy. Moreover, it has been demonstrated that US may be advantageous for tendon measurements, as it can provide a higher image resolution [5]. Compared with other imaging methods (e.g., MRI/CT), US appears advantageous due to safety, non-invasiveness and its possibility for real time assessment [3, 6]. In addition, US imaging also enables a visual assessment of hypoechogenicity, vascularity, and thickness. The technique may support the early identification of morphological changes in the musculoskeletal system, including muscles and tendons [7, 8]. US measurements may thus, be beneficial for pre- injury detection, rehabilitation management, and monitoring. Furthermore, US enables to assess micromorphological changes in the tendon’s structure. However, the technique has also drawbacks; for example, US images can be of low quality. It may be difficult to visually interpret the images because of speckle noise and difficulties in defining anatomical structures [5].

The PT has a conical shape, where the proximal part is wider, and then narrows until the final attachment on tibial tuberosity. Moreover, the tendon is not homogenous along it entire length and may have a different structure, including collagen content, collagen type, and extracellular matrix density [9]. From the physiological point, the proximal part of PT (approximately to 10 mm) might be affected by tensile loads and greater stress-strain relationship due to an impingement, in which the inferior patellar pole and the proximal posterior surface of PT compress on each other [10]. Due to high relationship of PT force-elongation [11], also the middle region of PT is exposed to overload. The middle region of PT (50% of the length)–approximately 20–25 mm from the apex patella, indicate that stress might be greater than at the distal portion of the tendon, and thus tendon stress is unlikely to be the main factor in the etiology of PT overloading [12].

Previous studies have evaluated the reliability of US in assessing PT thickness in healthy volunteers [13–17], subjects with suspected injuries and patients [18, 19] and track cyclists [20]. In those studies, intra- and inter-rater reliability was investigated using various statistical approaches, including intra class correlation coefficient (ICC) [13–17], standard error of measurement (SEM), minimal detectable change (MDC) [13], Bland-Altman plots [13–15, 17], analysis of variance [16] and correlation analyses [18]. Most of the previous studies evaluated PT thickness over a single location, placed 10 mm inferior to the patellar pole [13–17], even if more recent studies investigated PT thickness at multiple locations [17, 19–21]. Holm et al. [17] and Toprak et al. [21] evaluated PT thickness at 6 mm and 10 mm proximal to the patellar pole and tibial tuberosity. Klich et al. [20] more recently assessed PT thickness in track cyclists after competition at four locations, set at 5-10-15-20 mm inferior to the apex of the patella. This study reported greater differences between pre- and post-PT thickness at location placed 15 mm and 20 mm from the apex of the patella in sprint and endurance track cyclists. Finally, Castro et al. [19] made assessments at 25%, 50%, and 75% of the length of PT. In those studies, PT thickness was measured in the longitudinal view since Ekizos et al. [22] reported poor reliability for the measurement of PT’s cross-sectional area. Consequently, the current body of literature advocates for PT thickness over several locations even if the relative and absolute reliability has not been clearly reported.

Track cycling, especially sprint events, results in increased muscle tension and muscle soreness in the anterior thigh muscles [23–25]. Decreased sprint performance and greater fatigue can lead to the development of delayed-onset muscle soreness, decreasing sprint performance and affecting thigh musculature [23, 25]. An epidemiological study reported that cyclists can be exposed to knee overload injuries, especially PT overloading, which leads to anterior knee pain [26]. According to Klich et al. [20], acute fatigue in the middle region of PT, in track-cyclist athletes characterized by great quadriceps muscle mass, may cause hypoechogenicity, as one of the acute factors of fatigue and overloading [2].

Gaining a better understanding of the reliability of PT thickness may be useful in diagnosing acute fatigue-induced overloading by US imaging. We aimed to assess PT thickness at different locations of the tendon structure to identify changes in PT thickness at specific areas of the tendon. Therefore, the primary aim of the current study was to investigate the relative and absolute intra-rater and inter-rater reliability of the assessment of PT thickness at multiple locations in line with the GRRAS recommendations [27]. The secondary aim was to determine the differences in PT thickness between elite track cyclists and elite soccer players, with the latter group of individuals acting as a control to assess changes in PT thickness as a function of different sport specifics and training environment, e.g., isolated movement pattern on bike (track cycling) vs. multi-dimensional movement patterns on soccer fields.

## Materials and methods

### Participants

The population of the study included a group of 15 male elite track cyclists aged between 22 and 33 years (mean age (SD) of 26 (4) years, body height: 185.3 (8.6) cm, body mass: 85.5 (6.8) kg, body mass index (BMI): 25.3 (0.7) kg/m^2^) and 15 male elite soccer players aged between 22 and 27 years (mean age (SD) of 24 (2.5) years, body height: 183.3 (4.5) cm, body mass: 83.2 (5.4) kg, BMI: 23.3 (0.5) kg/m^2^). The track cyclists were members of the national team specializing in sprint events, with a mean training experience of 12.2 (2.3) years. All cyclists were competing in international-level track races, including the World Cup, European Championship, and World Championship. The soccer-player group included elite high division soccer players. The inclusion criterion for both groups was having trained for ≥10 years, while the exclusion criteria for both groups included (1) current or previous PT injuries or symptoms and (2) a history of surgery in the lower extremity. Soccer players were recruited for this study as controls to assess the differences between individual and team sports and between sprint and mixed performance sports. In our study, we evaluated only PT thickness in athletes without knee pain or current injury, who were considered asymptomatic subjects.

The expected effect size to assume differences in PT thickness in track cyclists and soccer player were estimated using G*Power software (version 3.1.9.2; Kiel University, Kiel, Germany) [28]. We calculated the power (1-β) for a two-way analysis of variance (ANOVA), by defining the sample size as 28, set a ‘large’ effect size (Cohen’s *f*) of 0.72, with the level of significance set to be *a* = 0.05, and a power of 0.90. The sample size calculation showed that at least 14 participants per group were necessary, but to account for potential dropouts, 15 participants were recruited. Furthermore, to estimate effect size for reliability analysis expressed by ICC, a sample size calculator performed by Arifin [29] was used. The sample size was defined as 30, set a minimum acceptable reliability (ICC) (ρ0): 0.60, with the level of significance set to be *a* = 0.05, and a power of 0.90. The sample size calculation showed that at least 15 participants per group were necessary. A total of 30 participants were recruited, and none of the participants were excluded from further investigation.

The study was approved by the Ethical Committee of the University Research Ethics Committee at the Wrocław University of Health and Sport Science (project identification code: 26/2016) and it was conducted according to the Helsinki Declaration. All subjects signed the written informed consent before participation in the study.

### Experimental procedures

Ultrasonography was performed using a Honda HS-2200 ultrasound scanner (Honda, Toyohashi, Japan) with a 7.5 (6.0 to 11.0) MHz and 40 mm linear array transducer (HLS-584 M, Honda, Japan) in grayscale B-mode. The settings of the US system were standardized for all participants and remained the same for all measurements [20]. In accordance with the methods described by Skou and Aalkjaer (14) the scan depth was set to 1.8 cm. Patellar tendon thickness was measured according to the recommendations of the European Society of Musculoskeletal Radiology [30].

**Fig 1:**
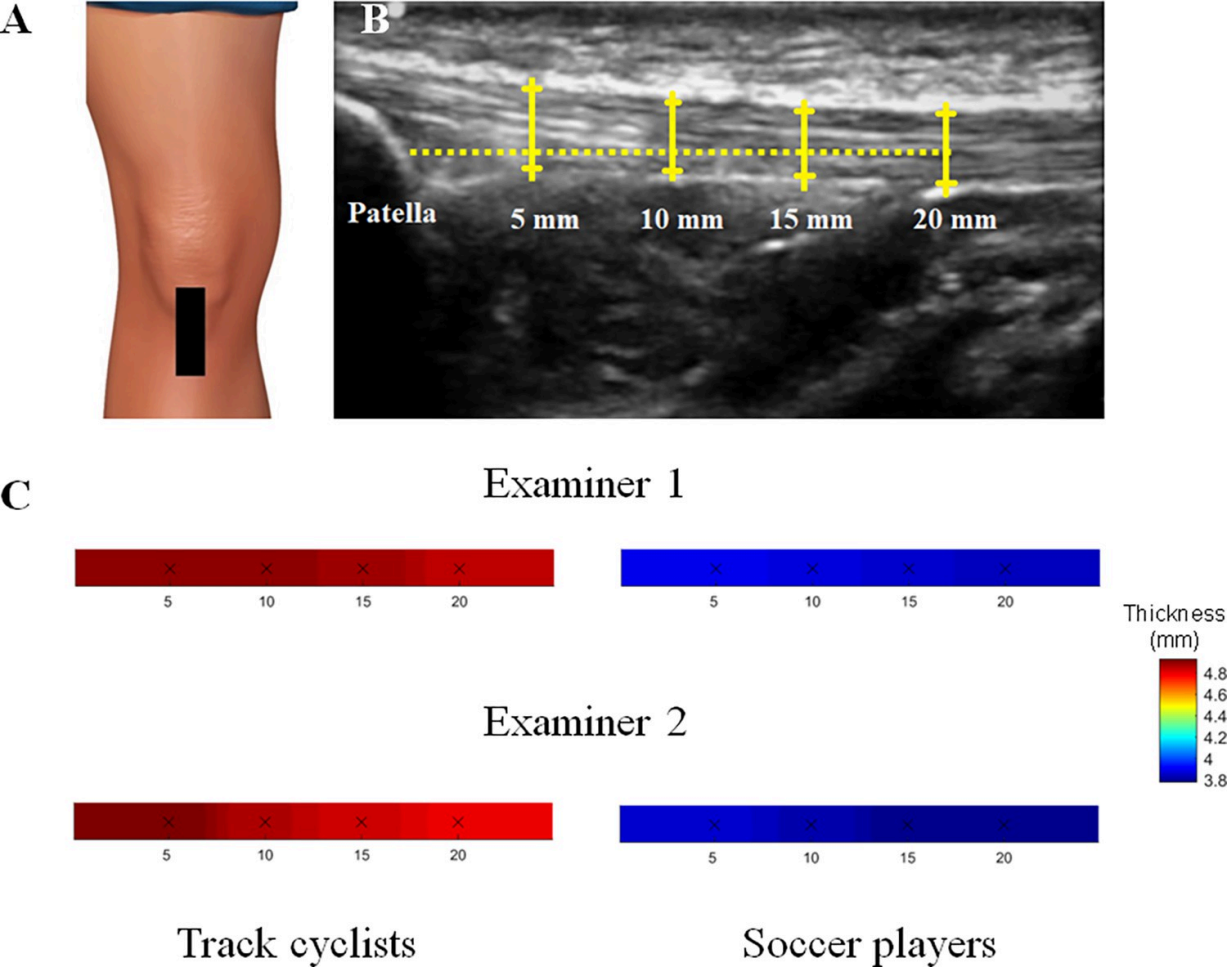
Ultrasound assessment and measurement of the patellar tendon. (A) the linear transducer placed longitudinally distal to the patella, (B) thickness of the PT was assessed at four locations: 5, 10, 15, and 20 mm inferior to the apex of the patella, (C) the 3D graphical representation of PT thickness at each location (5-10-15-20 mm).

The participants were lying in a supine position with their right knee flexed at approximately 30°. A pillow was placed under the popliteal space during the examination. This knee position prevents errors in measurements related to the concave position of the posterior thigh muscles and PT extension. During the scans, the linear transducer was placed longitudinally distal to the patella (Fig 1A). The thickness of the PT was assessed at four locations: 5, 10, 15, and 20 mm inferior to the apex of the patella. The tendon borders were defined inferiorly as the first hyperechoic region between the subcutaneous tissue and the deep fascia layer (Fig 1B) [20].

Ultrasound images of the PT were obtained by two sonographic examiners who specialized in musculoskeletal US and had ten (examiner 1) and twelve (examiner 2) years of clinical experience. Both examiners were physical therapists experienced (six and five years of experience) in lower extremity imaging. Examiners underwent 2 hours of training before conducting this study on the particular US settings and examination procedures (positioning of the subjects and transducer). The protocol was the same for all participants. All participants were asked to avoid physical activity for at least 2 days before the assessment. A screening evaluation of images of the PT was performed by an experienced orthopedic surgeon (specialized in knee disorders and injuries) to avoid signs of PT tendinopathy. The order in which the imaging locations were assessed was randomized, and the examiner was randomly selected for each assessment. The examiners were blinded to each other’s measurements and to the group of participants (track, cyclist, or soccer players). Each examiner took two US images of the PT thickness within a 10-minute period (test). After 30-minutes, the same procedure was repeated to obtain two new US images of the PT thickness (retest). After 1-week, both examiners measured the PT thickness from the US images of the test and retest session. The average of the PT thickness measures from each of the two images at test and retest were used for the statistical analyses in line with previous studies [14, 31].

The intra-rater reliability of PT thickness was calculated for each examiner and for each group (track cyclists, soccer players). The inter-rater reliability of PT thickness was calculated for each group (track cyclists, soccer players) from the images obtained by each examiner. The US images were randomly divided into blocks of 6 to decrease the potential learning effect. Maps of PT thickness were generated using inverse distance-weighted interpolation of the averaged values at each location (5-10-15-20 mm) to obtain a 3D graphical representation. The 3D images were obtained for graphical purposes using inversed distance interpolation by a factor of 2 with Franke and Nielson weightings [32]. Each map was standardized according to the body height of the subject (Fig 1C).

### Statistical analysis

Statistical analyses were performed using the statistical software SPSS 21 (SPSS Inc., Chicago, USA). Descriptive statistics (means ± standard deviation (SD) were calculated. Normality was evaluated using the Shapiro-Wilk normality test.

Intra- and inter-rater reliability was calculated according to the method described by Koo and Li [33] who estimated the two-way mixed effects, absolute agreement, and multiple raters/measurements (ICC_2, k_). The formula used was as follows:

$$ICC=\frac{{MS}_{R}-{MS}_{E}}{{MS}_{R}+\frac{{MS}_{C}-{MS}_{E}}{n}}$$

Where MSR = mean square across rows, MSW = mean square across the residual sources of variance, MSE = mean square error, MSC = mean square across columns, n = number of subjects, and k = number of raters/measurements.

Reliability was classified as poor (ICC<0.5), moderate (0.5<ICC<0.69), good (0.7<ICC<0.89) or excellent (ICC>0.9) [34]. The standard error of measurement (SEM = SDD/√2) and the minimal detectable change (MDC = SEM×1.96×√2) were calculated [35]. To determine the measurement precision, the limits of agreement (LOAs) were also calculated. The LOA was defined as the difference between the mean difference and the upper and lower LOAs [36]. Finally, two-way analysis of variance (ANOVA) followed by Tukey’s HSD post hoc test was used for groups (track cyclists and soccer players) and location (5, 10, 15, 20 mm) as independent factors to assess (1) the differences within groups and (2) differences between groups. Partial eta squared (η^2^) was calculated to assess the effect size. *P*-values <0.05 were considered statistically significant.

## Results

### PT thickness in track cyclists and soccer players

Table 1 provides the mean values, and Fig 1C shows the topographical maps of PT thickness at 5, 10, 15, and 20 mm in track cyclists and soccer players. The two-way ANOVA revealed a statistically significant group x location interaction effect on PT thickness for examiner 1 (*F* (5, 40) = 11.7, *p* = 0.001, η^2^ = 0.81) and examiner 2 (*F* (5, 46) = 13.8, *p* = 0.001, η^2^ = 0.78). The post hoc analysis showed that the PT thickness was larger for track cyclists compared with soccer players at all locations (*p*<0.001). Moreover, the within-group analysis for examiner 2 showed that the track cyclists had different thicknesses at 10 and 15 mm than at 20 mm and across individuals (*p*<0.001) and that the soccer players had different thickness values at 15 and 20 mm (*p*<0.001) (Table 1, Fig 1C).

**Table 1:** Ultrasound measurements for PT thickness (mm) at the four measurement points (5-10-15-20mm).

		Track cyclists (n = 15)	Soccer players (n = 15)	p-value**
		Mean (SD)	Mean (SD)	
**5 mm**	Examiner 1	4.90 (0.07)	3.90 (0.07)	<0.001
	Examiner 2	4.93 (0.08)	3.86 (0.05)	<0.001
**10 mm**	Examiner 1	4.90 (0.06)	3.88 (0.06)	<0.001
	Examiner 2	4.86 (0.04)*	3.83 (0.05)	<0.001
**15 mm**	Examiner 1	4.89 (0.06)	3.86 (0.03)*	<0.001
	Examiner 2	4.83 (0.05)*	3.79 (0.05)*	<0.001
**20mm**	Examiner 1	4.85 (0.07)*	3.84 (0.05)*	<0.001
	Examiner 2	4.80 (0.05)*	3.78 (0.04)*	<0.001

Abbreviations

* Significant differences within-groups compared to location placed at 5mm

** Significant differences between both groups (track cyclists vs. soccer players) (p≤0.05)

### Intra- and inter-rater ICC

Intra-rater reliability ranged from good to excellent (ICC_2,k_ from 0.75 to 0.96) in both groups. The ICCs for examiner 1 were good and excellent at all locations among the track cyclists (0.77 (95% CI = 0.32 to 0.92)– 0.94 (95% CI = 0.58 to 0.96)) and soccer players (0.79 (95% CI = 0.42 to 0.90)– 0.92 (95% CI = 0.7 to 0.95)). The intra-rater ICCs for examiner 2 were slightly higher than those for examiner 1 among the track cyclists; the ICCs of examiner 2 ranged from 0.75 (95% CI = 0.45 to 0.94) to 0.96 (95% CI = 0.68 to 0.98). Among the soccer players, we found slightly lower ICCs for examiner 2 than for examiner 1, ranging from 0.79 (95% CI = 0.35 to 0.85) to 0.90 (95% CI = 0.69 to 0.92). Inter-rater reliability was good and excellent among the track cyclists (from 0.75 (95% CI = 0.49 to 0.92) to 0.9 (95% CI = 0.57 to 0.94)) and soccer players (from 0.76 (95% CI = 0.44 to 0.84) to 0.89 (95% CI = 0.63 to 0.90)) (Table 2).

**Table 2:** Intra-class correlation coefficients (ICC), 95% confidence Interval (95% CI), standard error of measurement (SEM), minimal detectable change (MDC) and 95% limit of agreement (LOA) for intra- and inter-rater reliability of patellar tendon thickness.

Measure		Track cyclists (n = 15)				Soccer players (n = 15)			
		5 mm	10 mm	15 mm	20 mm	5 mm	10 mm	15 mm	20 mm
**Intra-rater**									
Examiner 1	ICC	0.77	0.84	0.89	0.94	0.79	0.83	0.89	0.92
	95% CI	0.32–0.92	0.41–0.94	0.62–0.94	0.58–0.96	0. 42–0.90	0.45–0.92	0.62–0.95	0.7–0.95
	SEM [mm]	0.05	0.03	0.02	0.02	0.04	0.04	0.03	0.03
	MDC [mm]	0.12	0.10	0.07	0.06	0.10	0.10	0.08	0.07
	LOA [mm]	1.4	1.3	0.8	0.7	1.0	0.8	0.6	0.6
	95% CI	-0.4–1.5	-0.6–1.4	-0.3–1.1	-0,2–0.9	-0.5–1.4	-0.6–1.2	-0.5–0.9	-0.2–0.11
Examiner 2	ICC	0.75	0.87	0.92	0.96	0.79	0.85	0.89	0.90
	95% CI	0.45–0.94	0.5–0.96	0.71–0.93	0.68–0.98	0.35–0.85	0.56–0.90	0.53–0.94	0.69–0.92
	SEM [mm]	0.05	0.03	0.03	0.02	0.04	0.04	0.03	0.02
	MDC [mm]	0.11	0.08	0.07	0.04	0.10	0.10	0.06	0.05
	LOA [mm]	1.5	1.1	0.7	0.7	1.0	0.9	0.6	0.6
	95% CI	-0.3–1.6	-0.3–1.2	-0.1–1.0	-0.2–0.10	-0.2–1.2	-0.3–1.2	-0.3–1.0	-0.2–1.0
**Inter-rater**	ICC	0.75	0.82	0.87	0.90	0.76	0.82	0.85	0.89
	95% CI	0.49–0.92	0.6–0.92	0.55–0.93	0.57–0.94	0.44–0.84	0.72–0.86	0.66–0.95	0.63–0.90
	SEM [mm]	0.04	0.03	0.03	0.03	0.04	0.04	0.03	0.03
	MDC [mm]	0.11	0.10	0.09	0.08	0.10	0.09	0.08	0.08
	LOA [mm]	1.4	1.2	0.8	0.8	1.1	0.9	0.7	0.7
	95% CI	-0.1–1.6	-0.4–1.4	-0.3–1.0	-0.3–1.3	-0.3–1.3	-0.4–1.0	-0.3–1.2	-0.1–0.12

### Intra- and inter-rater SEM, MDC, and LOA

In general, the intra-rater SEMs were higher among track cyclists than among soccer players, mostly due to larger thickness values. For examiner 1, the SEMs were below 0.05 mm among track cyclists and 0.04 mm among soccer players. Examiner 2 was more precise in measuring PT thickness in both groups. The inter-rater SEMs ranged from 0.03 to 0.04 mm in both groups. The intra-rater MDCs ranged from 0.05 mm to 0.12 mm, whereas the inter-rater MDCs ranged from 0.08 mm to 0.11 mm (Table 2).

The intra-rater LOA ranged from 0.6 mm to 1.5 mm, with the soccer player group showing lower values. The LOA for intra- and inter-rater reliability showed a mean difference that represented less than 1.5 mm of the corresponding thickness (Table 2).

## Discussion

The current study showed that the relative intra- and inter-rater reliability of measuring PT thickness with US imaging ranged from good to excellent at four different locations in track cyclists and soccer players. The reliability expressed in relative (ICC) and absolute terms (SEM and MDC) was highest at the area located 20 mm from the patellar apex. Moreover, the PT was thicker among track cyclists than among soccer players. Our study revealed the best location in relation to the apex of the patella enabling more reliable measurement of PT thickness. Moreover, this study was the first to report values in asymptomatic two groups of athletes enabling to compare findings with findings from clinical studies addressing PT injury mechanisms. These findings might be useful in monitoring PT thickness across several locations due to its conical structure.

Previous studies reported good to excellent (ICC 0.7 to 0.93) relative inter-rater reliability for PT thickness [13–15] measured 10 mm distal to the inferior patellar apex. We also obtained good to excellent inter-rater ICCs, particularly at the distal area located 20 mm from the patellar apex in track cyclists and soccer players. Moreover, our study investigated for the first time the reliability of measuring PT thickness at four different locations. The relative intra-rater reliability was in agreement with prior studies assessing PT thickness at 10 mm from the patellar apex in asymptomatic subjects [13–15, 17]. The PT thickness has also been assessed in locations more distal to the tibial tuberosity [19, 21]. Castro et al. [19] reported that the middle region of the PT (50% of the length) was the easiest region to define the tendon, while the distal region (75% of length) was more difficult to access. In our study, we found excellent intra- and inter-rater reliability at the location 20 mm inferior to the apex of the patella. Furthermore, our results were similar with Castro et al. [19], because the middle region of the PT was located at a distance of approximately 20 mm from the apex. Toprak et al. [21] reported that intra-rater reliability was higher at the distal location. On the contrary, Holm et al. [17] observed higher inter-rater reliability at the distal insertion point. Most previous studies have reported the intra- and inter-rater reliability of measuring PT thickness at a single location [13–16, 18, 37]. In general, PT thickness is measured at the widest hypoechoic area [18], or at 10 mm from the patellar apex [13–16]. Only the study by Fredberg et al. [37] reported measurements at intervals of 5 mm, i.e., at 10, 20, 25, and 30 mm inferior to the patellar apex, and showed that PT thickness is significantly larger in proximal areas than in distal areas. Moreover, those authors have not reported relative or absolute reliability to confirm their results contrary to the GRRAS recommendations [27].

Only one previous study investigated the absolute reliability (SEM and MDC values) of measuring PT thickness [13]. In the study absolute reliability was also calculated. These authors reported an SEM of 0.05 mm and MDC of 0.32 mm in asymptomatic subjects, which were slightly higher than those obtained in our study (SEM: 0.03 mm- MDC: 0.09 mm to 0.10 mm). It should be noted that the lowest SEM and MDC values were observed for the most distal location (20 mm). In our study, we have shown that athletes have thicker PT than that soccer players and thicker than the values previously reported for asymptomatic adults [14]. Our measurement precision values were similar to those previously reported by Skou and Aalkjaer (14) and Holm et al. [17] (LOA 0.7 mm to 0.9 mm) and smaller than those reported by O’Connor et al. [16].

The changes in thickness and relative and absolute reliability were most likely related to the conical structure of the PT and the potential difficulty of clearly defining the tendon borders [2, 19, 21]. In the examiner’s clinical experience, PT borders can be difficult to define proximally relative to the tibial tuberosity but are more easily identified distally (15–20 mm from the apex). Our results suggest that PT thickness, measured at different tendon areas, can play a crucial role in the clinical evaluation of athletes and patients with patellar tendinopathy. The clinical validity of multisite measure of PT thickness evaluated in athletes should be addressed taking idiopathic mechanisms of patellar tendinopathy in consideration, e.g., assessing quadriceps cross sectional area and force-deformation relationship of the tendon [38]. Moreover, it is important to mention that reliability may differ among injured and/or symptomatic populations. The proposed multisite assessment can also be used for monitoring changes in PT thickness during the treatment of acute and chronic knee pain disorders. Similarly, the early identification of changes in thickness throughout the PT may also be important for athletes of different sports to prevent overloading injuries. The differences between track cyclists and soccer players in PT thickness might be related to training load and demands of the sports [39]. Sprint track cyclists represent a group of athletes whose training is focused on strength training and short-duration repetitive sprint exercises [23]. This type of training might lead to hypertrophy of the quadriceps. In line with the previous statement, Rønnestad et al. [40] reported that strength training affects the tendon cross-sectional area and consequently leads to changes in the structural and mechanical properties of the PT. Thus, the PT becomes thicker, stiffer, and larger in terms of the cross-sectional area as a result of strength training [41].

Ultrasonography is commonly recommended for examination of the structure of the PT, including its stiffness in patients with tendinopathy and overloading injuries [42]. Investigations of the structure of the PT among athletes may provide important clinical findings regarding morphological properties and consequently, injury mechanisms. Changes in the thickness of PT in athletes have not been investigated or described in previous studies. In our study, we observed a thicker PT in track cyclists than in soccer players (approximately 25% in all locations). Comparing other populations, observed a 14.6% thicker PT in junior female volleyball players than in asymptomatic subjects. Of note, these differences were larger than the calculated SEM and MDC, highlighting the importance of the practiced sport. Concerning the practical implications of this study, measuring thickness at four locations, could be used to monitor potential risk of PT injuries as the loading of the tendon is not completely homogeneous, optimize rehabilitation process by detecting eventual thinner or thicker PT. All in all, assessing the PT thickness at multiple locations may improve return to sport after tendon injury. The use of this approach by medical staff (physicians, physical therapists and athletic coaches) and sport scientists can contribute to monitor PT thickness loading over time and prevent risks of overloading injury in PT.

Finally, we recognize that the current study has some limitations. First, since the PT can reach 45 mm, more distal points could be assessed in future studies. We decided to assess these four points since they are the most common sites of symptoms in patients with patellar tendinopathy. Second, we included asymptomatic track cyclists and sports players, i.e., individuals without symptoms in the knee. We do not currently know whether the PT reliability results would be similar in injured athletes or patients. Third, we could have reported the relative (%) measurement locations rather than the absolute location (mm). Future studies should include symptomatic adults (both males and females) and larger sample sizes. Finally, US imaging is an operator-dependent measurement tool, as examiners with more experience yield higher reliability [43].

## Conclusions

In conclusion, US has been demonstrated to be a reliable method of assessing the thickness of the PT. The middle region of the PT (corresponding to 15 mm and 20 mm from inferior to the apex of the patella) was revealed to be the most reliable point to assess PT thickness. The PT was thicker among track cyclists than among soccer players, with larger differences at 15 mm from the apex. Of note, these differences were larger than the SEM and MDC, underlining the changes in PT thickness mostly related to training load and type of sport. Our findings on absolute and relative reliability provide important normative data that can be used in the future in longitudinal studies [44].
